# SimBA: A methodology and tools for evaluating the performance of RNA-Seq bioinformatic pipelines

**DOI:** 10.1186/s12859-017-1831-5

**Published:** 2017-09-29

**Authors:** Jérôme Audoux, Mikaël Salson, Christophe F. Grosset, Sacha Beaumeunier, Jean-Marc Holder, Thérèse Commes, Nicolas Philippe

**Affiliations:** 1 SeqOne, IRMB, CHRU de Montpellier -Hopital St Eloi, 80 avenue Augustin Fliche, Montpellier, 34295 France; 2 Institute of Computational Biology, Montpellier, 860, Rue Saint-Priest, Montpellier Cedex 5, 34095 France; 3 University Lille, CNRS, Centrale Lille, Inria, UMR 9189 - CRIStAL - Centre de Recherche en Informatique Signal et Automatique de Lille, Lille, F-59000 France; 4 University Bordeaux, Inserm, BMGIC, U1035, Bordeaux, 33076 France

**Keywords:** RNA-Seq, Transcriptomics, Benchmark, Pipeline optimization

## Abstract

**Background:**

The evolution of next-generation sequencing (NGS) technologies has led to increased focus on RNA-Seq. Many bioinformatic tools have been developed for RNA-Seq analysis, each with unique performance characteristics and configuration parameters. Users face an increasingly complex task in understanding which bioinformatic tools are best for their specific needs and how they should be configured. In order to provide some answers to these questions, we investigate the performance of leading bioinformatic tools designed for RNA-Seq analysis and propose a methodology for systematic evaluation and comparison of performance to help users make well informed choices.

**Results:**

To evaluate RNA-Seq pipelines, we developed a suite of two benchmarking tools. *SimCT* generates simulated datasets that get as close as possible to specific real biological conditions accompanied by the list of genomic incidents and mutations that have been inserted. *BenchCT* then compares the output of any bioinformatics pipeline that has been run against a *SimCT* dataset with the simulated genomic and transcriptional variations it contains to give an accurate performance evaluation in addressing specific biological question. We used these tools to simulate a real-world genomic medicine question s involving the comparison of healthy and cancerous cells. Results revealed that performance in addressing a particular biological context varied significantly depending on the choice of tools and settings used. We also found that by combining the output of certain pipelines, substantial performance improvements could be achieved.

**Conclusion:**

Our research emphasizes the importance of selecting and configuring bioinformatic tools for the specific biological question being investigated to obtain optimal results. Pipeline designers, developers and users should include benchmarking in the context of their biological question as part of their design and quality control process. Our *SimBA* suite of benchmarking tools provides a reliable basis for comparing the performance of RNA-Seq bioinformatics pipelines in addressing a specific biological question. We would like to see the creation of a reference corpus of data-sets that would allow accurate comparison between benchmarks performed by different groups and the publication of more benchmarks based on this public corpus. *SimBA* software and data-set are available at http://cractools.gforge.inria.fr/softwares/simba/.

**Electronic supplementary material:**

The online version of this article (doi:10.1186/s12859-017-1831-5) contains supplementary material, which is available to authorized users.

## Background

### RNA-seq technology: power and versatility

The unprecedented evolution of next-generation sequencing (NGS) technologies in transcriptomics (RNA-Seq) has shaped computational biology and facilitated new advances in genomic medicine [1]. RNA-Seq examines the dynamic nature of the cell’s transcriptome, the portion of genome that is actively transcribed into RNA molecules which allows researchers to determine when and where genes are turned on or off in a variety of cell types and circumstances.

RNA-Seq offers benefits in the detection of novel transcripts as it does not require context-specific probes rendering it more flexible than microarrays that cannot be modified to reflect evolving requirements. However, for RNA-Seq to evolve beyond the realm of analytic research to clinical use, performance benchmarking of the RNA-Seq-specific bioinformatic tools are needed to ensure accuracy and reproducibility [2].

### RNA-seq analysis process: dependence on the biological question

Given the increasing variety of biological questions being investigated using RNA-Seq analysis [3], the community has developed a broad range of bioinformatic tools to address specific needs. This growing selection of tools should, in theory, make it possible for users to design highly optimized pipelines that deliver best results for any specific biological question. The traditional process of the RNA-Seq analysis [4] consists of a multi-step pipeline - starting with mapping, followed by the quantification of known transcript or gene and finally statistical analysis of the expression including differential expression analysis and clustering. RNA-Seq can also be used to identify new genes and transcript variants including splice junctions, SNVs, indels or gene fusions predictions that are of particular relevance in the study of cancer [5].

An important consideration in analyzing RNA-Seq is that, due to the complexity of interpretation, pipelines must be optimized to the biological question being investigated to yield meaningful results. Some analyses require more sensitivity while others put emphasis on precision. Other factors such as the sequencing protocol and compute resources available also have to be considered. However, despite all the work done in optimizing specific tools, few people have sufficient understanding of their characteristics to make informed choices on the best ones to include in a given pipeline, or how they should be configured. To make matters worse, the number of distinct permutations of pipeline components and configurations creates a real combinatorial challenge.

One strategy that helps the researcher address this problem is the systematic evaluation of software alternatives based on a set of simulated reads. For instance, in a recent benchmarking study [6], the authors assessed RNA-Seq alignment tools on three human and malaria datasets at the read, junction and base levels. However, their benchmark suffered from limitations that could impact their conclusions. We question the relevance of the metrics used in their comparisons: the way multiple alignments are discarded (those do not use the standard NH tag in the SAM file), the fact that highly-expressed junctions have more weight in the junction-level analysis than under-expressed ones, and the fact the the authors rely solely on the CIGAR string to assess the alignment (while some ambiguous alignments might have distinct CIGAR strings). The authors use the dataset with the highest mutation rate of 3% to tune the parameters., so RNA-Seq benchmarking efforts to-date have tended to provide a review of the pipeline tools available at a given time, with a focus on individual components such as aligners [7–10], variant callers [11–13] or quantification methods [7, 14].

Other comparison strategies are based on modeling rules and computing metrics from real data sets of reads [15, 16] as it is assumed that simulating NGS reflecting the biological complexity and technical biases associated with sequencing is impossible. Recently, an original approach, TEASER, was introduced [17] to compare genomic aligners in a dynamic and flexible manner using a platform with a simple SaaS application to generate reports automatically. However, TEASER is not applicable to RNA-seq analysis. It can be complicated to transpose a specific benchmarking result to another biological context and to use results to optimize configuration a selected pipeline. As a result, many researchers rely on well known, highly publicized pipelines using standard configurations that may be ill-adapted to examining their particular biological question. As an example, it is not uncommon to see researchers using, for instance, TopHat2 with default parameters^1^. We believe that SimBA will make it easier to conceive benchmarks adapted to a specific biological question.

### RNA-seq challenge: The need for standard data-sets and benchmarking methodology

Combining a set of tools to create a complete analysis pipeline requires expertise and familiarity with the characteristics of each candidate software component of the workflow. This has implications for researchers and clinicians alike, as using the right pipeline, correctly configured, limits false positives and ensures that relevant genomic events are surfaced. This emphasizes the need for a benchmarking methodology that provides concrete information on the pipeline and configuration settings that yield best results for a given biological question.

Traditional simulation approaches like *BEERS* [10] or *Flux Simulator* (Flux) [18] aim to model RNA-Seq experiments *in silico* by sequencing reads from a reference genome according to annotated transcripts and by modeling different experimental behaviors. *BEERS* is an easy-to-use simulator of simplified reads, whose main weakness is that it does not take into account the preparation of the library that precedes the sequencing itself (fragmentation, RT-PCR, etc.). *Flux* provides a more realistic view as it models the wet-lab process and the type of sequencer and simulates sequence errors using techniques based on heuristic models. *Flux* also benefits from excellent documentation and a high configurability of each step of the simulation. However, though it does an excellent job in simulating the expression profile, library preparation and sequencing, it does not take into account the underlying biological significance of the reference transcriptome (genome modifications, cancerous cells, etc.). Other approaches to simulating cancerous data directly in sequenced reads using BAMSurgeon [19], rather than modifying a reference genome and its annotations.

Many of these approaches do not allow tools to be benchmarked in the context of a specific biological question [10]. This drives users to develop new methods based on their own private data-sets. Some initiatives have emerged such as Dream Challenge that provide sets of data for benchmarking but without the curation that provides test data relevant to each requirements that would enable consistent and reproducible comparisons.

## Implementation

**Fig. 1:**
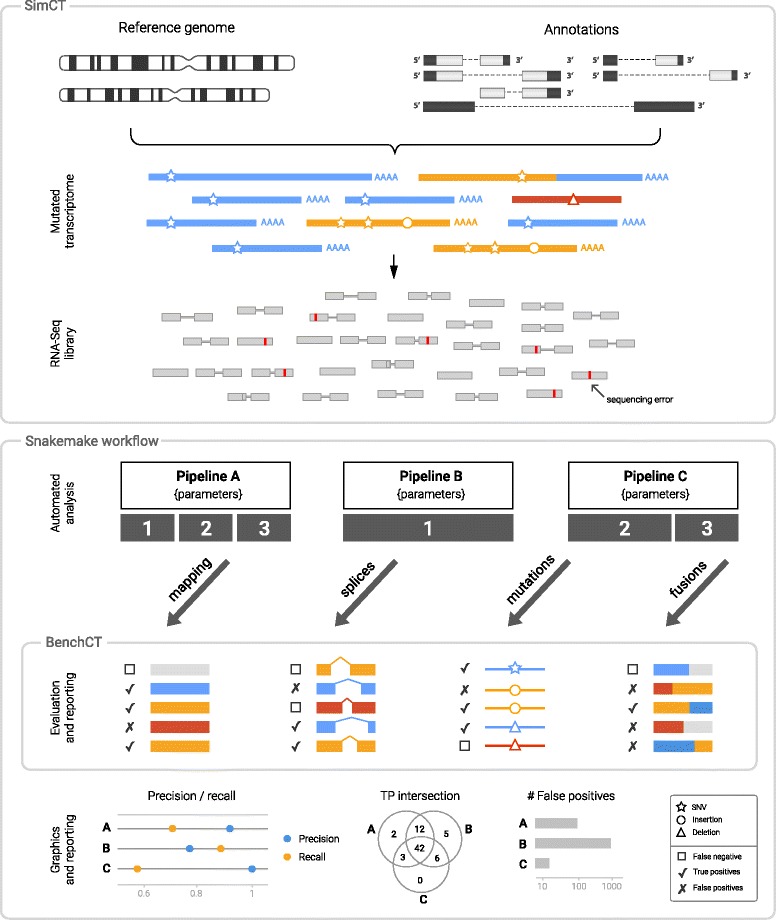
Overview of the SimBA benchmarking procedure. A benchmarking pipeline implemented with *SimBA* is composed of three components, i/ Simulation of synthetic data using *SimCT*, ii/ Processing of the synthetic data using a pipeline manager (i.e Snakemake [20], iii/ Qualitative evaluation of the results using *BenchCT*

We have developed *SimBA* (Simulation & Benchmarking Analysis), a software suite designed to evaluate the performance of an entire RNA-Seq pipeline in the context of a specific biological question. *SimBA* provides an integrated environment (see Fig. 1) that lets the users generate benchmark reports that evaluate the workflow of their choice. It is comprised of two components:

SimCT A configurable generator of simulated RNA-Seq data that can emulate specific biological mechanism (*ie.* SNVs, indels, fusions) and provide robust data sets covering cases such as fusion genes (or fusions). BenchCT A qualitative evaluation tool which assess any pipeline results against a simulated dataset to obtain a clear understanding of its performance characteristics in answering a particular biological question. In this paper, we illustrate how this vision applies in genomic medicine by building a set of RNA-Seq data simulations of a somatic condition and propose an automated workflow using Snakemake [20] to compare tools in three qualitative RNA-Seq use cases: SNVs, indels and fusion genes. The Snakefile used to generate all results presented in the article is available at https://github.com/jaudoux/simba-publication-pipeline.

### SimCT: RNA-Seq operation

*SimCT* is a complete and modular workflow to simulate RNA-Seq data from a reference genome and known transcript annotations.

**Fig. 2:**
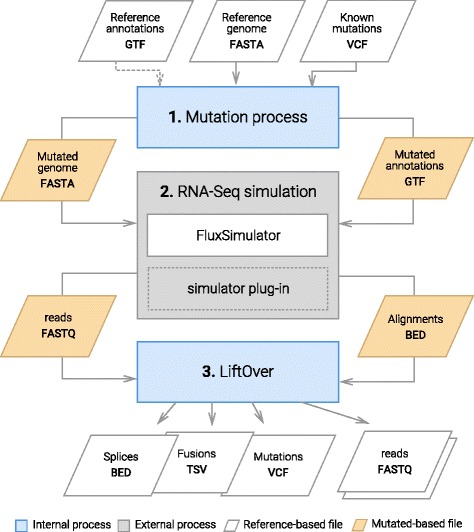
SimCT method. SimCT uses a reference FASTA and GTF annotations as input. A first process is intended to introduced biological variations in this reference to create a mutated reference. This new reference is then transfered to FluxSimulator, in order to generate an RNA-Seq experiment. Finaly FluxSimulator output are post-processed to transfer the coordinates from the mutated genome to the original reference

*SimCT* works in three steps (see Fig. 2):

1. The first step introduces a set of variants in the reference genome. *SimCT* then generates a haploid mutated genome in FASTA format and a set of GTF annotations whose coordinates are converted for this modified reference.
2. We then process the modified reference files using *FluxSimulator*, that offers good performance in generating a random expression profile and simulating reads by reproducing a complete *“in silico”* RNA-Seq protocol.
3. In the final step, the reads and corresponding alignments produced by *FluxSimulator* are sent to post-processing where alignment coordinates are converted to the coordinates of the original reference genome. The errors are then extracted from read sequence (encoded in lowercase by *FluxSimulator*) and a new FASTQ file is produced with alignments and errors encoded in the read name, similar to the approach in RNF [21].

#### Mutation process

Single nucleotide variants (SNVs) or insertion and deletions (indels) are introduced in the reference genome either randomly or guided by a reference VCF file (only biallelic sites are supported for the moment). Both mutation sources can be used together with – –vcf-ratio option, that defines the ratio of mutations taken from the VCF file provided, to those generated randomly. Overlap between mutations is forbidden.

##### Random mutations

Random mutations are generated given a defined rate per chromosome with equal probability for each of the four bases. The lengths of indels is chosen randomly between 1 and the max indel length (default is 15).

#### Gene fusions

Gene fusions (fusions) are generated by randomly picking two exons from the annotations, and combining their two parent genes into a fusion gene. This is accomplished by merging the upstream part of the parent gene of one exon with the downstream part of the parent gene of the other exon. Both fused exons are then merged to force the fusion junction between the two ends. The new fusion gene is saved in a separate FASTA file, and annotations of the fusions transcripts are added to the GTF file.

#### Read name format

*SimCT* includes the alignment position and errors of the simulated reads in their names, making it easier for developers to identify bugs or algorithm flaws in their alignment software. The read format is defined as :

$$read\_id:(chr,(-)pos,cigar(;)?)+:base64(err\_pos?) $$

The read name is composed of three components: (i) the read id from 0 to *n* *b*_*r* *e* *a* *d* *s*−1, (ii) a list of SAM-like [22] read alignments (chr, starting position and cigar chain), (iii) the positions of sequencing errors (if any) encoded in base64. For paired-end reads, a single read name is generated for both reads by using a single *r* *e* *a* *d*_*i* *d*, concatenating the alignments and merging the positions of sequencing errors.

#### Dataset characteristics

In addition to the FASTQ file containing reads with their alignments, *SimCT* also saves splice and fusion junctions and mutations supported by the sequenced reads in separate files. Fusion junctions are defined as either *non colinear junctions* (junctions on distinct chromosomes, or in reverse order), or *colinear junctions* with a junction length longer than a pre-defined threshold (300kb by default). This means, that colinear fusion junctions are not necessarily produced by the fusions introduced, but can also result from very large splice junctions of unfused genes.

### BenchCT: RNA-Seq qualitative analysis assessment

To assess the RNA-Seq analysis results based on simulated data, we have developed *BenchCT*. It loads the reference “truth database” of known dataset characteristics and compares it to the output files (SAM, BED, VCF, software specific files) of the pipelines being evaluated. For each characteristic, *BenchCT* classifies predictions as TP (true positives), FP (false positives) or FN (false negatives). We then compute two metrics for qualitative analysis: (*p* *r* *e* *c* *i* *s* *i* *o* *n*=*T* *P*/(*T* *P*+*F* *P*) and *r* *e* *c* *a* *l* *l*=*s* *e* *n* *s* *i* *t* *i* *v* *i* *t* *y*=*T* *P*/(*T* *P*+*F* *N*)). *BenchCT* uses genomic interval-trees available as *CracTools-core modules*, useful for quickly checking whether a position is at a given distance of the known position in the “truth database”.

#### Evaluation procedures

**Fig. 3:**
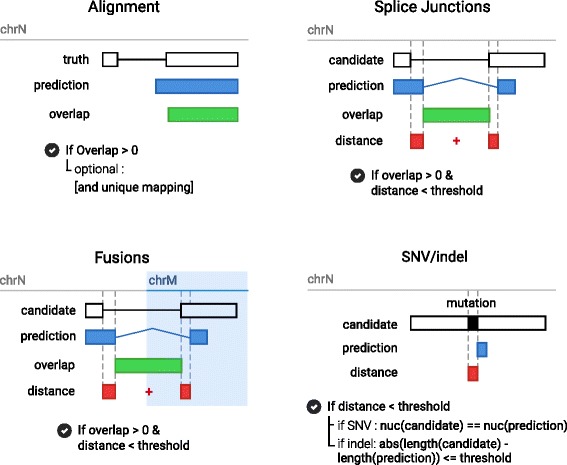
BenchCT evaluation procedures. Each event is evaluated with benchCT with a specific procedure that allow approximate matching. For alignement, only overlap between the prediction and the truth is evaluated. For Splice junctions and Fusions we expect an overlap between the prediction and a candidate in the truth database with a limited agreement distance according to the threshold. For mutation (SNV and Indel), similar procedure is used, as well as the verification of the mutation. For SNVs we evaluate the mutated sequence and for insertions and deletions, the length of the mutation

With *BenchCT*, we propose standardized procedures for the binary classification of alignments, splice junctions, indels, SNVs and fusions predictions. For each of these events, we have proposed a means of comparison of results with the “truth database” (see Fig. 3). In the case where several alignments can correctly be made, it may be possible to find multiple event/mutation scenarios that explain the simulated data-set. In such cases, *BenchCT* attempts to score these solutions as correct using a heuristic approach.

##### Alignment evaluation

*BenchCT* alignment evaluation is performed as follows: if a proposed alignment overlaps the real alignment by at least one base (encoded in the read name), it is considered as true. Parameters can be set to ignore some alignments (*ie*. multi-mapped reads) that are counted as false positives (FP). If multiple alignments are found for the same read, only the first one encountered is evaluated and the others ignored.

##### Splice junction evaluation

*BenchCT* defines splice junctions as genomic intervals (or GI).

$$GI = (chr, start, end, strand) $$

True splice junctions are loaded in a GI-tree. This data structure, built with a set of interval-trees for each chromosome strand, can later be queried against a splice junction predicted by an analysis pipeline to find overlapping true splice junctions. An evaluated splice prediction *p*, defined as a GI, is then searched in the GI-tree and any resulting overlapping GIs are then included in the set *C* of candidates. For each candidate *c*∈*C* we compute the distance

$$d(p,c) = |p_{start} - c_{start}| + |p_{end} - c_{end}| $$

If the distance *d* is less or equal than the threshold defined (default value is 10), the splice is considered true.

If multiple predictions correspond to the same candidate, only the first is counted as a TP and the others ignored. If multiple candidates match a prediction, we select the one having the minimum distance (arg min_*c*_ *d*(*p*,*c*)). The same treatment applies to indels, SNVs and fusions.

##### Indels and SNVs evaluation

The mutations are evaluated with the same GI procedure were *start* and *end* positions are equal, and the strand is left undefined. The GI *p* used to search the GI-tree is defined as

$$p = (p_{chr},p_{pos} - threshold, p_{pos} + threshold) $$

For indels we evaluate the distance between the predicted indel length and the observed one according to the threshold. For SNVs we also verify the validity of the predicted mutated nucleotide. The default threshold for mutations is 5.

##### Fusions evaluation

Fusions are evaluated in the same way as the other GI-based evaluations; fusion intervals are defined as (*c* *h* *r*_1_,*p* *o* *s*_1_,*s* *t* *r* *a* *n* *d*_1_,*c* *h* *r*_2_,*p* *o* *s*_2_,*s* *t* *r* *a* *n* *d*_1_) and transformed into regular GI with *c* *h* *r*=*c* *h* *r*_1_·*c* *h* *r*_2_, *s* *t* *a* *r* *t*=*p* *o* *s*_1_, *e* *n* *d*=*l* *e* *n* *g* *t* *h*(*c* *h* *r*_1_)+*p* *o* *s*_2_, *s* *t* *r* *a* *n* *d*=*s* *t* *r* *a* *n* *d*_1_·*s* *t* *r* *a* *n* *d*_2_. The default distance threshold for fusions is set to 20.

### Simulated dataset

Simulated dataset used in the results were generated using the primary assembly of the version *GRCh38* of the human genome. Annotations were downloaded from *Ensembl* FTP (version 86) in GTF format. SNVs were introduced at a rate of 0.0014 and 0.0016 (‘-s‘ option) for *normal* and *somatic* condition respectively. Insertions and deletions were introduced at a rate of 0.0001 (‘-d‘ and ‘-i‘ options) in both conditions with max indel length set to default (15). *SimCT* was used with – –vcf-file and – –vcf-ratio 0.95 options to randomly take 95% of the mutations from the VCF file of *common SNP* provided by dbSNP (version 20160527). Finally 100 fusions (number chosen arbitrarily) were introduced for the *somatic* condition. This does not mean that all of the fusions genes are expressed and found in the RNA-Seq data as this depends on the expression profile randomly generated by *FluxSimulator*.

Expression profiles were generated using the following parameters for *FluxSimulator*: *k*=−0.7,*x*_0_=15,000,25,000 for normal and somatic conditions respectively, $x_{1} = x_{0}^{2}$, NB_MOLECULES =10^6^. NB_MOLECULES is the number of expressed transcript molecules. Parameters *k* and *x*_0_ are part of a mixed power and exponential law of the expression profile, where *k* is the exponent of the rank of gene expression. This means that the smaller *k* the smaller the decay of the expression resulting in highly expressed transcripts having closer expression levels. *x* and *x*_1_ govern the parameter of the exponential decay by multiplying the rank of gene expression ^*k*^. This mostly affects the tail of the gene expression spacing the differences further out between lowly expressed genes. Parameter ranges were estimated for mammalian cells by nonlinear fitting to expression levels observed in experimental results by the developers of *FluxSimulator* [18] and were chosen accordingly in the suggested ranges. For a more detailed explanation we refer to the paper and webpage of *FluxSimulator*.

Two data-sets were generated for each of the conditions (normal and somatic), varying the length of the reads (101bp and 150bp) for a given sequencing depth of 160 million paired-end reads. The fragment length was set to 250bp with a standard deviation of 50bp and initial biological material was set 10 million molecules. Finally we provided *FluxSimulator* a custom error model calibrated on Illumina Hiseq 2500 data retrieved from SRA (SRR1611183), mapped with GEM mapper [23] and calibrated with *FluxSimulator* ‘-t errormodel’ option.

### Tools and parameters

#### Mutation discovery pipelines

Twelve mutation discovery pipelines were defined:

1. Three based on STAR mapping; (1) STAR with Freebayes, (2) STAR with GATK (HaplotypeCaller), and (3) STAR with SAMtools (mpileup and bcftools).
2. Three with Hisat2 mapping; (4) Hisat2 with Freebayes, (5) Hisat2 with GATK (HaplotypeCaller), and (6) Hisat2 with SAMtools (mpileup and bcftools).
3. Three with Hisat2_2pass mapping; (7) Hisat2_2pass with Freebayes, (8) Hisat2_2pass GATK (HaplotypeCaller), and (9) Hisat2_2pass SAMtools (mpileup and bcftools).
4. One using CRAC mapping software (10) that integrates its own calling algorithm.
5. Two “meta-calling” pipelines that are the union of the results of two pipelines using bcftools merge command; (11) A combination of STAR-GATK and CRAC variants, and (12) A combination of STAR-GATK and Hisat2_2pass-GATK.

Mapping software settings and versions are described in the Table 1, they are identical for all datasets. Hisat2_2pass consists in running Hisat2 a second time on the same dataset using splice junctions discovered at the first pass.

**Table 1:** Software versions and parameters used to generate the results

Software	Version	Parameters
HISAT2	2.0.4	–max-intronlen 300000 –novel-splicesite-
		outfile {output.novel_splice}
HISAT2_2PASS	2.0.4	–max-intronlen 300000 –novel-splicesite-
		infile {input.novel_splice}
		–novel-splicesite-outfile {output.novel_splice}
STAR	v2.5.2b	–twopassMode Basic –alignMatesGapMax
		300000
		–alignIntronMax 300000
STAR_fusion	v2.5.2b	–twopassMode Basic –alignMatesGapMax
		300000
		–alignIntronMax 300000 –chimSegmentMin
		–chimJunctionOverhangMin 12
		–chimSegmentReadGapMax 3
		12 –alignSJstitchMismatchNmax 5 -1 5 5
CRAC	2.5.0	-k 22 –detailed-sam –no-ambiguity –deep-snv
CRAC_fusion	2.5.0	-k 22 –detailed-sam –no-ambiguity –deep-
		snv –min-chimera-score 0
GATK	1.3.1	-T HaplotypeCaller -dontUseSoftClippedBases
		-stand_call_conf 20.0
		-stand_emit_conf 20.0
FREEBAYES	1.0.2	*default*
SAMTOOLS mpileup	1.3.1	*default*

The variant calling pre-processing procedure has been adapted from GATK best practices [24]. For all mapping procedures we applied the following procedure:

1. Mark duplicates with *PicardTools*.
2. Apply *SplitNTrim* (GATK command) procedure which splits reads into exon segments (removing Ns but maintaining grouping information) and hard-clip any sequences overhanging into the intronic regions.
3. Apply *ReassignOneMappingQuality* (GATK command) read filter to reassign all good alignments to the default value of 60.

This re-calibration pipeline produced a BAM file used by the variant calling software whose versions and settings are described in Table 1. For the CRAC pipeline we have used the cractools extract command that produces a VCF file from a BAM file produced with CRAC.

#### Gene fusions discovery pipeline

For gene fusions we ran special versions of CRAC and STAR called CRAC_fusion and STAR_fusion that are described in the Table 1. Hisat2 was not included here, since it does not support fusion discovery. STAR_fusion and CRAC_fusion parameters were set to classify as a fusion, colinear junctions having a distance superior to 300kb identical to *SimCT* fusions definition.

STAR_fusion was run with the —chimSegmentMin option varying from 10 to 30 (10, 12, 14, 16, 18, 20, 22, 24, 26, 28, 30).This option controls the minimum mapped length of the two segments that is allowed. The fusions detected by STAR are listed in the file called Chimeric.out.junction that contains one line per chimeric alignment. These alignments were summarized based on the chimeric breakpoints, and filtered-out to remove chimeric alignments not defined at the base level (field 8>=0) and further filtered with a minimum recurrence threshold of 2.

The fusions from CRAC were extracted from the BAM file using cractools extract and further filtered with a minimum recurrence threshold of 2. We then created multiple versions of CRAC_fusion by filtering fusions based on their *chim_values* from 0 to 1 (0, 0.2, 0.4, 0.6, 0.8, 0.85, 0.9, 0.95, 1). This options controls the algorithmic quality of chimeric junctions detected by CRAC based on a machine learning procedure [25].

### Evaluation and reporting

#### BenchCT parameters

*BenchCT* was run using default threshold parameters described in “Evaluation procedures” section. For alignments we used the option “max_hits” to limit evaluation to alignments having the SAM’s “NH” field equal to 1.

#### Reporting

The automatic reporting procedure includes an precision/recall plot for each evaluated event, displaying these two metrics (computed by *BenchCT*) along with the Fscore that is used to order the pipelines.

$$Fscore = 2\times \frac{recall \times precision}{recall + precision} $$

*BenchCT* was parametrized to output true positives (TP) that were used to build intersection plots with Upset [26] R package.

## Results

Our objective was to create a use-case relevant to genomic medicine by creating realistic simulated RNA-Seq data-sets and using them to compare performance of twelve state-of-the-art pipelines. Our benchmarks determined the ability of each pipeline to detect biological events relevant to the needs of genomic medicine.

### A corpus for medicine genomxic

We generated four sets of RNA-Seq data relevant to genomic medicine, with documented characteristics that correspond to specific biological questions, in order to provide a consistent and reproducible basis for the evaluation of pipelines.

The four data-sets were designed to address the sequencing of human samples in two biological contexts; normal and somatic cells. The normal condition contains a genomic layer of polymorphisms with rates close to the observations of 1000 genomes [27]. To improve our simulation quality, 95% of introduced mutations were taken from common human polymorphisms [28]. The somatic condition contains higher mutation rates, a more complex gene expression profile and gene fusions.

Two data-sets were generated for each of these conditions, varying the length of the reads (101bp and 150bp) and using a sequencing depth of 2x80 million paired-end reads. These two lengths were chosen to mimic the *Illumina Hiseq 2500* sequencing platform which typically produces these read lengths. The deep sequencing was used to create a base dataset that can be further sub-sampled for other applications.

The Table 2 is a summary of the four data-sets that constitute our genomic medicine corpus; a detailed description of *SimCT* and *FluxSimulator* parameters is described in “Simulated dataset” section of the method.

**Table 2:** Summary of the data-sets characteristics

Dataset name	Read size	SNV	Insertion	Deletion	Splice	Colinear	Non colinear
						fusion	fusion
*GRCh38-101bp-160M-normal*	2×101	28554	1747	1908	94904	73	0
*GRCh38-150bp-160M-normal*	2×150	28625	1754	1915	94855	64	0
*GRCh38-101bp-160M-somatic*	2×101	47139	2564	2716	132511	99	14
*GRCh38-150bp-160M-somatic*	2×150	47577	2648	2771	134235	108	15

### Aligner selection

The first step in the bioinformatic analysis process is alignment. For this reason, we selected three aligners for the benchmark; (i) CRAC [29] for its high precision, (ii) STAR [30] for its sensitivity and, (iii) Hisat2 [31] which offers a good compromise between precision and recall (Additional file 1: Figures S2 and S3).

We then evaluated the predictions of twelve variant calling pipelines based on these three aligners and three variant callers (see “Mutation discovery pipelines” section). Then, we used *SimBA* to optimize mapping software predictions for gene fusions by independently tuning their parameters (see “Gene fusions discovery pipeline” section).

In the following, we discuss only part of results, by selecting representative dataset and evaluation metrics. Complete results are available as supplementary material.

### Benchmarking performance in SNV detection

**Fig. 4:**
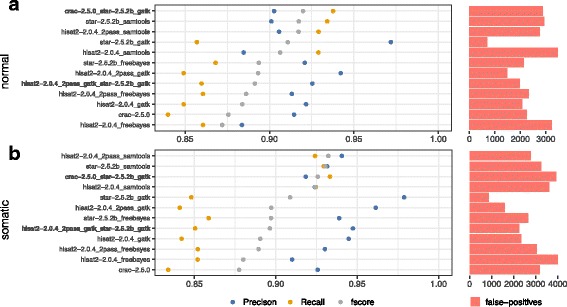
Precision and recall of SNV calling. **a** SNV precision/recall in *GRCh38-150bp-normal* data-set. **b** SNV detection in *GRCh38-150bp-somatic* data-set

This benchmarking resulted in the following observations for SNV detection (see Fig. 4).

Performance in identifying SNVs varies significantly depending on the pipeline. An example of this is the STAR-GATK pipeline which is the most accurate (> 97*%*) while all other pipelines provide precision of between 90 and 95%. The SAMtools [32] caller has a much higher recall than GATK [33] and Freebayes [34] regardless of the aligner (∼ 92*%* vs ∼85*%*).

We observed significant differences in calling pipeline performance between “normal” (Fig. 4a) and “somatic” (Fig. 4b) data-sets. For example, Hisat2_2pass-GATK results vary from 91 to 94% of precision between normal and tumoral data-sets (datasets A and B), implying that there is background noise that is progressively smoothed by the addition of new real biological events.

Performance differences also occurred when different variant calling procedures are used with the same aligner. If we consider the example of STAR-SAMtools vs STAR-GATK in Fig. 4a, we obtain results of 90%/93% versus 97%/86% in term of precision/recall.

Performance was heavily impacted by the configuration settings of any given tool. For example, when Hisat2 is run in two-pass mode, precision is improved (+5% on average) but processing time (∼ 1*h*10 with 10 CPUs) double (Additional file 1: Figure S1).

Our experiments show that the size of the reads has no discernible impact on the SNV results delivered by the pipelines which allowed us to focus our attention on the 150bp data-sets (Additional file 1: Figure S4).

**Fig. 5:**
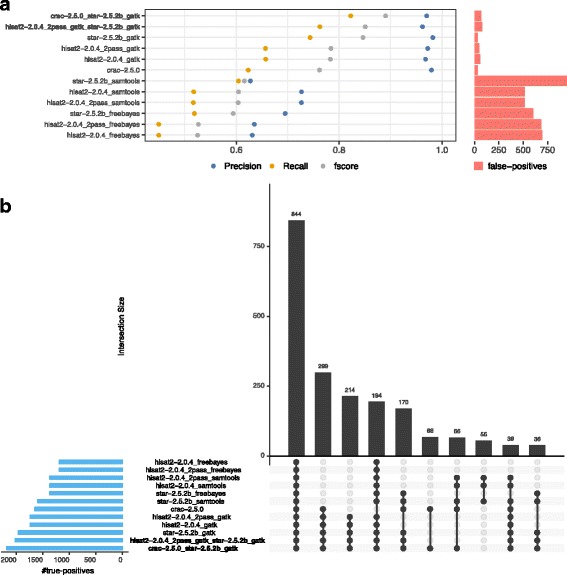
Precision and recall of indel calling. **a** Insertion precision/recall in *GRCh38-150bp-somatic*. **b** Intersections of true positives insertions found by calling pipelines in the *GRCh38-150bp-somatic* data-set

We also discovered that the performance of the pipelines can be significantly improved by combining certain complementary tools. The meta-calling pipelines were defined based on the true positive intersections (ex: Fig. 5b) to maximize the recall for both SNVs and indels. As an example, in the Fig. 4b, CRAC and STAR-GATK identify each ∼ 4000 true SNV’s which allows a gain in recall from 85% for both tools independently to 93% when they are combined, while maintaining excellent precision. It must be stressed that all the pipelines are not necessarily complementary. For example, the meta-pipeline Hisat2-GATK + STAR-GATK perform worse than the “simple” STAR+GATK pipeline (ex: Fig 4b similar recall but precision decreases from 98 to 95%).

### Benchmarking performance in detecting insertions and deletions

The insertion and deletion benchmarks displayed the same tendencies on all four data-sets (Additional file 1: Figures S5 and S6); In general, CRAC is very accurate (> 99*%* precision with fewer than 10 false positives per data-set), STAR-GATK is very sensitive (> 75*%* recall) and Hisat2_2pass-GATK provides a good precision/recall compromise (*F* *s* *c* *o* *r* *e*∼0.8). We selected the *GRCh38-150bp-160M-somatic* data-set for insertion calling in Fig. 5.

In all cases the meta-pipeline CRAC+STAR-GATK produced best results with ∼ 85*%* recall, ∼ 97*%* precision and an *F* *s* *c* *o* *r* *e*>0.9. We observed the same trend with the other “meta-pipeline” Hisat2_2pass-GATK+STAR-GATK, as we did in the case of SNVs; sensitivity was scarcely better than STAR-GATK while precision was lower.

To illustrate this performance difference between the pipelines, let us consider the number of actual deletions identified by each; STAR-GATK identified 2091 deletions, Hisat2_2pass-GATK+STAR-GATK performed slightly better with 2125 more true positives (+ 1pt) while CRAC+STAR-GATK performed substantially better identifying 2295 (+ 10pts).

A key point is that by combining outputs of complementary tools, it is possible to improve recall without precision loss as can be seen in the case of the CRAC+STAR-GATK combination. The Freebayes caller seems less well suited to RNA-Seq using default parameters with less precision and recall than the two other callers. A final point to consider is the impact of the biological event being examined on the performance between the benchmarked tools. For example, the Hisat2_2pass-SAMtools pipeline, though well suited to detection of SNVs performs poorly in detecting indels with precision that falls from 94 to 80% and recall that falls from 92 to 54% (Fig. 4b vs Fig. 5a).

### Benchmarking performance in detecting gene fusions

In this third use-case, we examined fusion detection and, specifically, the detection of reads with a chimeric junction.

**Fig. 6:**
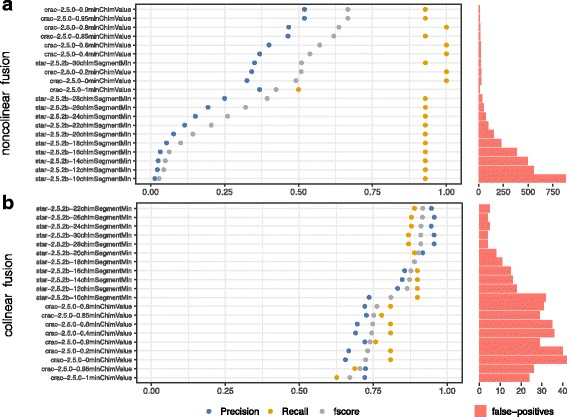
Precision and recall of gene fusion detection. Evaluation of gene fusions detection pipelines on the *GRCh38-101bp-160-somatic* dataset. Fusions were splited in two category with an individual evaluation. **a** Colinear fusion where the fusion involves to genomic locations that are located on the same strand of the same chromosome with a distance superior to 300kb. **b** non-colinear fusions wich does not satisfy the *colinear* criteria

Many studies on the subject have been published [35–37] but none have fully addressed the problem of alignment associated with this type of event. This is significant as incorrect or ambiguous alignment can, by itself, generate substantial numbers of false fusions. We selected two mappers optimized for the detection of fusions; CRAC with the integration of a model based on machine learning [25] and STAR that recently added a procedure to identify reads associated with fusions. We varied two parameters in these tools, CRAC’s *chim_value* and STAR’s *chim_segment*. Figure 6 confirms that these two parameters have tremendous impact on precision related to fusion detection (especially those that are non-colinear).

Observing the influence of the cutoff parameter on *Fscore*, there is a direct correlation (Fig. 6a); the greater the value of STAR’s *chim_segment* or CRAC’s *chim_value*, the more the precision increases (rising from 0 at 10% of precision for a chim_segment varying from 10 to 30). This underlines the importance of testing candidate pipelines with different cutoff values against a reference data-set to identify the best settings to address any given biological question.

It is however difficult to declare STAR or CRAC better suited for our simulated data-sets as somatic data-sets have a very low number of true fusions to realistically simulate the biological question (see Table 2). Indeed, between the 101bp and the 150bp data-sets performances of STAR and CRAC are very different. STAR, with 30 chim_segment, yields a better *Fscore* (0.65) than CRAC (0.25) with a 0.85 chim_value (see Fig. 6). It is very unlikely that these differences are due to the length of sequences, but rather to the nature of fusions in each data-set (see Additional file 1: Figures S7 to S8).

We also observe that colinear fusion predictions are more accurate than in the case of non-colinear fusions for both aligners, (see Fig. 6 a and b).

Nevertheless, sensitivity for both types of fusions is high when using both CRAC and STAR with an average value of between 80% and 90% of fusions detected on all data-sets examined.

In conclusion, it can be said that the principal challenge in exploring fusions is to correctly configure the tools for the specific data-set being examined as this significantly reduces the number of false-positives produced during the alignment phase. Indeed, eliminating false chimeric alignments at the very first step of a fusion gene detection pipeline is much safer than using empirical filters based on annotations later on, as true fusions could be incorrectly filtered out.

## Discussion

### Limitation of benchmarked tools studies

Numerous benchmarking studies of RNA-Seq analysis tools have since been conducted the appearance of RNA-Seq. The problem is that each study relies on its own simulated data-sets as a basis for the comparison of tools rendering comparisons between tools evaluated in different studies difficult. The methodology used to assess how close the predictions are to the truth also varies from one study to another. A further source of difficulty is that the tools themselves are constantly being upgraded making it virtually impossible to obtain a reliable, updated view of the performance of all the available tools on the market at a given moment in time. This means that it is impossible to reliably compare performance figures between studies to draw conclusions on the best pipelines to use to answer a specific biological question. As an example, the RGASP project [8] only represents a picture of benchmarked tools at the time of the study, though most of the tools benchmarked have been upgraded since. Such difficulties underline the need for a benchmark methodology that can reflect results of latest software versions.

We believe that in order to have an accurate picture of performance of available tools, the community must have access to a corpus of realistic simulated data-sets that emulate various biological conditions. The impact of results on the variant calling (see “Benchmarking performance in SNV detection” section) according to the mutation rate of the input data-set illustrates the need to have standardized reference data-sets that allow an equitable, accurate benchmarking of all tools according to a biological context.

### Need a solution to benchmark tools

Because the examination of some biological questions require more sensitivity while others require higher precision or must be capable of running using limited computational resources, users need a solution that offer possibility to choose the right bioinformatic components for an analysis depending on the specific biological question and computational environment available.

To illustrate this, our study relies on two standard cases of biological questions; variant calling and gene fusion detection. The proposed approach changes the focus of bioinformatic benchmarking from evaluations of discrete algorithmic components to an evaluation of a complete pipeline’s ability to provide meaningful answers to genomic medicine problems. This facilitates the evaluation of new tools by the community fostering more rapid innovation in this field.

Even if an original approach based on this principle [17] was announced, enabling users to quickly compare genomic aligners in a dynamic and flexible manner, the proposed approach is only dedicated to the benchmark of genomic aligners. Thus, to the best of our knowledge, there is no modular, interactive solution to benchmarking RNA-Seq software.

### SimBA a modular benchmarking workflow

We have designed *SimBA*, as two independent components, *SimCT* and *BenchCT*, that were conceived in a modular fashion with the objective of extending their features and scope of relevance.

*SimCT*’s modular design makes it possible to include other read simulators to handle emerging sequencing technologies such as “Oxford Nanopore” or “PacBio” sequencers via a “plug-in” architecture. A similar “plug-in” approach to profile simulators has also been adopted. *SimCT* is not restricted to RNA-Seq as WGS or exome sequencing simulators could be integrated to produce multi-omics analysis benchmarks. The modularity of *SimCT*, makes it easy to extend it in the near-term to the sequencing of multi-clonal tumoral transcriptomes, where a sample contains a heterogenous mixture of cancer cell clones that have emerged from a common ancestor.

*BenchCT* is a complete solution, featuring an easy installation, advanced algorithms, fast execution, multi-core architecture support. *BenchCT* is also highly configurable thanks to its YAML setup file. *BenchCT* was designed to provide an easy way to benchmark tools with interpretable metrics, sensitivity and precision. However, we plan to incorporate the evaluation of expression estimates into *SimBA* as the community defines more standardized comparison metrics [7, 14]. In future, we also intend to include other qualitative evaluation cases, such as transcript reconstruction.

## Conclusion

We propose *SimBA*, an open benchmarking solution that can be used to generate simulated data-sets with high configurability as well as a means to evaluate the performances of analysis pipelines as they relate to various qualitative questions such as read alignment, variant calling and gene fusion detection.

In this paper, we have created a means for comparison of results related to questions relevant to genomic medicine by producing reference data-sets that mimic cancerous cells and the investigation of important events in this type of pathology such as mutations, indels and fusions. The results described in our paper emphasize the need for a benchmarking tool that can: *(i)* Simulate reference data-sets emulating a specific biological context, *(ii)* Calibrate a pipeline for a data-set specific to a biological question, *(iii)* Aggregate and compare results of a variety of bioinformatic tools to explore how the combination of various tools impacts the overall performance.

By contrast to the existing work on the RNA-seq analysis, we focused on the necessity of proposing a flexible and integrated benchmarking suite that helps users optimize their workflows for biological questions. *SimBA* gives a standard analysis methodology and benchmark data-sets which guarantee a high level of accuracy and reproducibility.

## Availability and requirements

- **Project name:** SimBA;
- **Project home page:** http://cractools.gforge.inria.fr/softwares/simba/;
- **Operating systems:** Linux/MAC;
- **Programming language:** Perl;
- **Other requirements:** Perl5, cpanm, Flux Simulator, cractools-core;
- **License:** MIT

## Endnote

^1^ A preliminary search on Google Scholar with “Tophat2 ‘default parameters”’ returns about 1000 results compared to 4,500 for TopHat2 alone

## Additional file

Additional file 1 Supplemental Materials: SimBA: A methodology and tools for evaluating the performance of RNA-Seq bioinformatic pipelines. (PDF 259 kb)

## Abbreviations

BAM: Binary sequence alignement/map (SAM) format
BED: Browser extensible data format
Flux: FluxSimulator
FN: False negative
FP: False positive
Fusion: Gene fusion
GI: Genomic interval
GI-tree: Genomic interval-tree
GTF: Gene transfer format
Indel: Insertion and deletion
NGS: Next generation sequencing
NA-Seq: RNA-Sequencing
SaaS: Software as a service
SNV: Single nucleotive variant
TP: True positive
VCF: Variant call format
YAML: YAML ain’t markup language
WGS: Whole genome sequencing
