# Supplementary material for: SimBA: A methodology and tools for evaluating the performance of RNA-Seq bioinformatic pipelines
Source: BMC Bioinformatics. 2017 Sep 29;18:428. doi: 10.1186/s12859-017-1831-5 (PMC5623974; doi:10.1186/s12859-017-1831-5)
Supplement: Additional file 1 — Supplemental Materials: SimBA: A methodology and tools for evaluating the performance of RNA-Seq bioinformatic pipelines. (PDF 259 kb) [file 12859_2017_1831_MOESM1_ESM.pdf]

Supplemental Materials: A methodology and tools for evaluating the performance of RNA-Seq bioinformatic pipelines

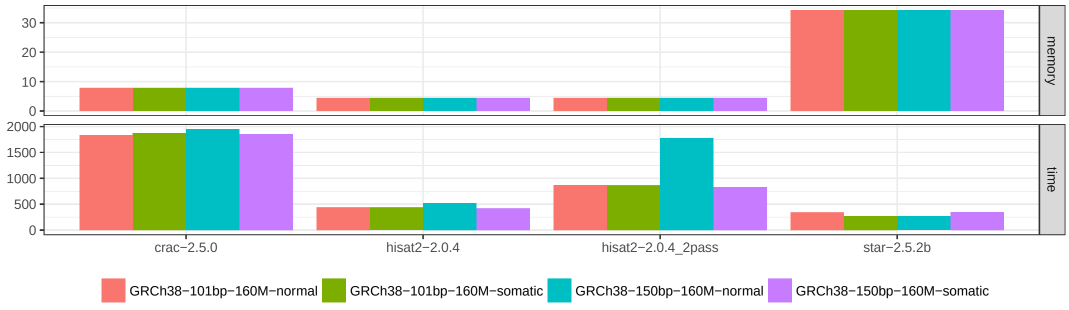

Supplementary Figure 1: Memory and time of mapping softwares
Performances where measured with unix /usr/bin/time command. Mappers were run with 10 cores. Time is set to *user* time (in minutes), while memory is set to *max memory usage* (in Gb).

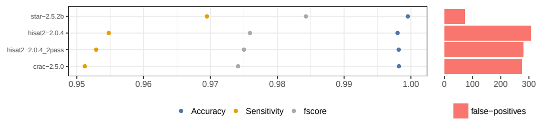

(a) GRCh38-101bp-160M-normal

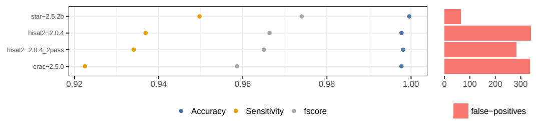

(b) GRCh38-101bp-160M-somatic

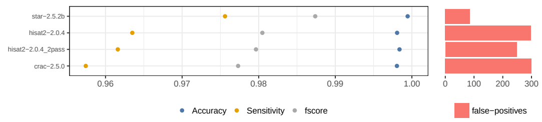

(c) GRCh38-150bp-160M-normal

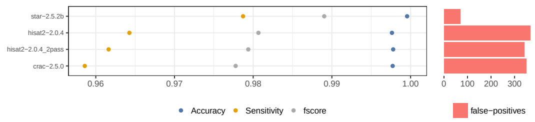

(d) GRCh38-150bp-160M-somatic

Supplementary Figure 2: Accuracy sensitivity for alignments

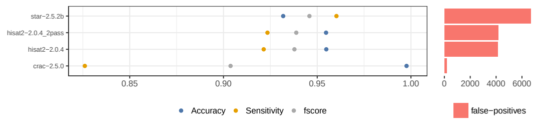

(a) GRCh38-101bp-160M-normal

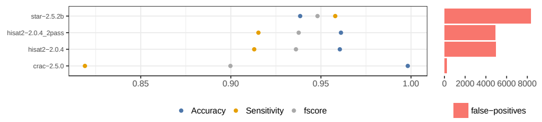

(b) GRCh38-101bp-160M-somatic

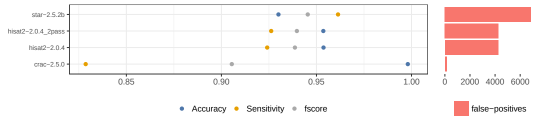

(c) GRCh38-150bp-160M-normal

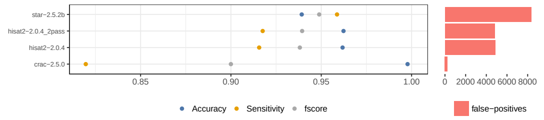

(d) GRCh38-150bp-160M-somatic

Supplementary Figure 3: Accuracy sensitivity for splices

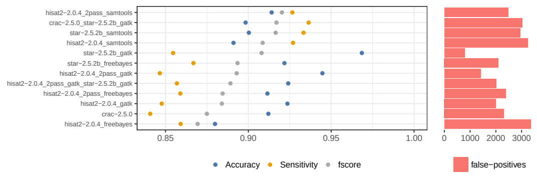

(a) GRCh38-101bp-160M-normal

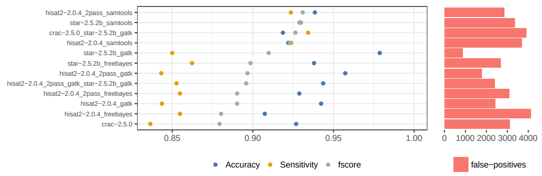

(b) GRCh38-101bp-160M-somatic

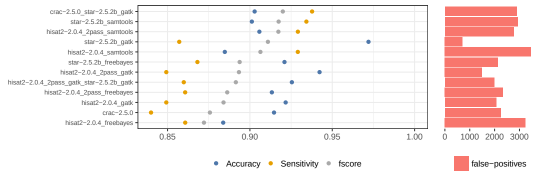

(c) GRCh38-150bp-160M-normal

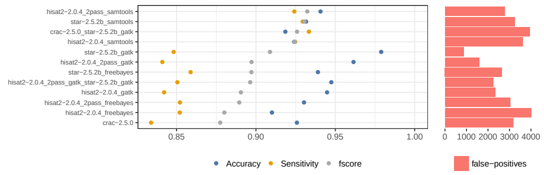

(d) GRCh38-150bp-160M-somatic

Supplementary Figure 4: Accuracy sensitivity for snps

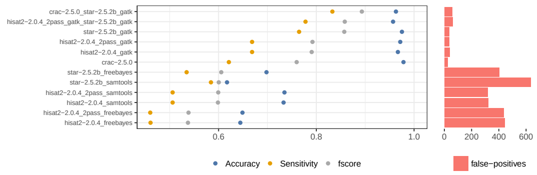

(a) GRCh38-101bp-160M-normal

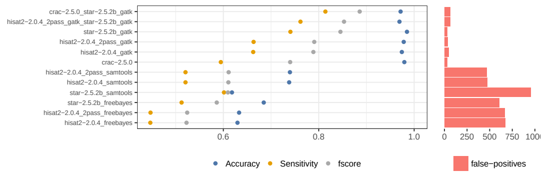

(b) GRCh38-101bp-160M-somatic

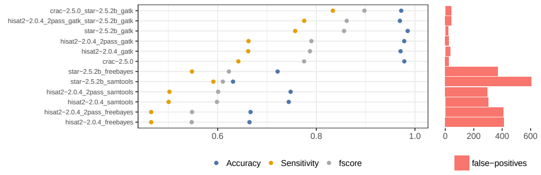

(c) GRCh38-150bp-160M-normal

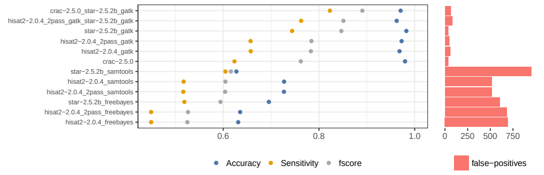

(d) GRCh38-150bp-160M-somatic

Supplementary Figure 5: Accuracy sensitivity for insertions

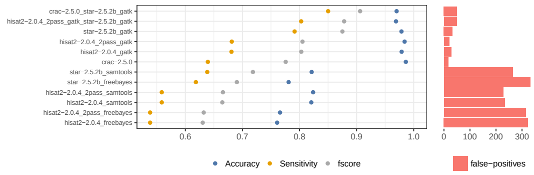

(a) GRCh38-101bp-160M-normal

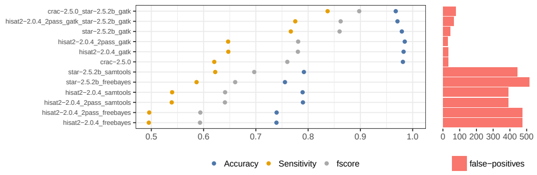

(b) GRCh38-101bp-160M-somatic

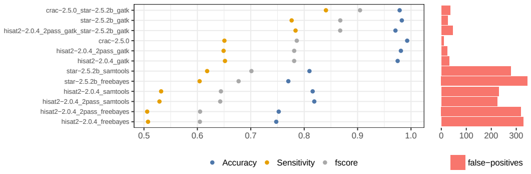

(c) GRCh38-150bp-160M-normal

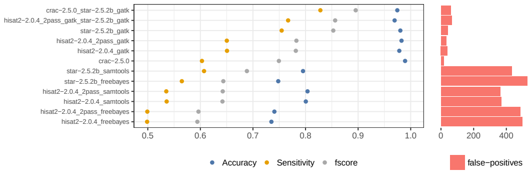

(d) GRCh38-150bp-160M-somatic

Supplementary Figure 6: Accuracy sensitivity for deletions

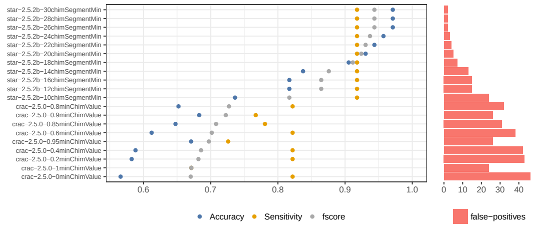

(a) GRCh38-101bp-160M-normal

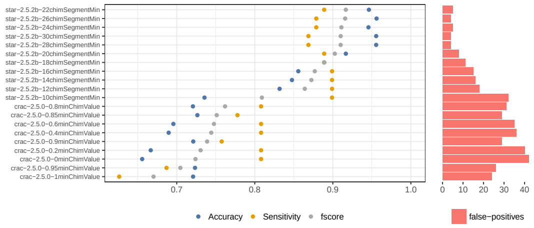

(b) GRCh38-101bp-160M-somatic

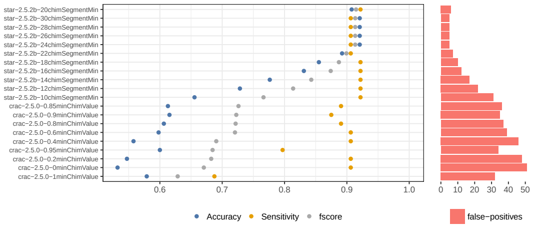

(c) GRCh38-150bp-160M-normal

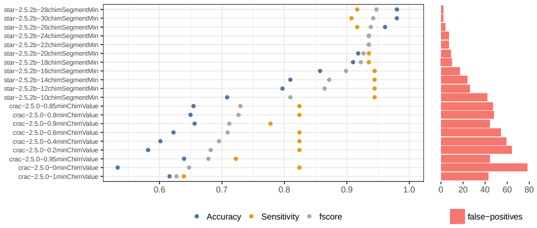

(d) GRCh38-150bp-160M-somatic

Supplementary Figure 7: Accuracy sensitivity for colinear_fusions

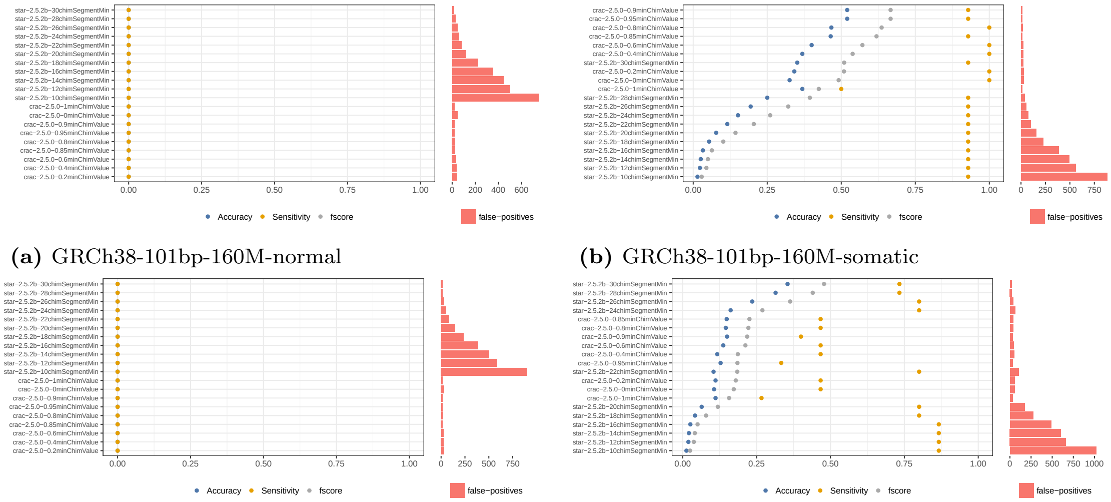

(a) GRCh38-101bp-160M-normal

(b) GRCh38-101bp-160M-somatic

(c) GRCh38-150bp-160M-normal

(d) GRCh38-150bp-160M-somatic

Supplementary Figure 8: Accuracy sensitivity for noncolinear_fusions
